# Timing From Diagnostic Imaging to Rotator Cuff Repair Does Not Significantly Impact Patient-Reported and Clinical Outcomes

**DOI:** 10.7759/cureus.117472

**Published:** 2026-10-05

**Authors:** Drew Dalby, Nathan Li, Mahdi Mazeh, Joshua Castle, Jared M Mahylis, Stephanie Muh

**Affiliations:** 1 Department of Orthopedic Surgery, Henry Ford Health System, Detroit, USA

**Keywords:** acute, chronic, mri, rotator cuff tear, ultrasound

## Abstract

Background

The decision to pursue rotator cuff repair (RCR) for symptomatic rotator cuff tears is multifactorial. While prior studies have evaluated predictors of operative versus nonoperative success, the impact of timing from diagnostic imaging to surgery on postoperative outcomes remains unclear. This study aimed to determine whether time from magnetic resonance imaging (MRI) or ultrasound to RCR affects clinical and patient-reported outcomes.

Methods

A retrospective cohort study was conducted of patients undergoing RCR. Patients were stratified into two cohorts based on time from imaging to surgery: <3 months and ≥3 months. Demographics, preoperative and postoperative visual analog scale (VAS) pain scores, Patient-Reported Outcomes Measurement Information System (PROMIS) scores, and active and passive range of motion were assessed. A subgroup analysis compared traumatic and nontraumatic tears. Complication rates were evaluated between groups.

Results

A total of 487 patients were included (342 <3 months; 145 ≥3 months). At one-year follow-up, VAS pain scores were significantly lower in the <3-month cohort (2.7 ± 2.7 vs. 3.3 ± 3.3; p = 0.05); however, this did not meet the minimal clinically important difference (MCID) of 1.4. No significant differences were found in abduction (p = 0.44), forward flexion (p = 0.57), or external rotation (p = 0.35). Overall complication rates were similar (17.3% vs. 20%; p = 0.43), as were re-tear (9.4% vs. 11.7%; p = 0.43), adhesive capsulitis (5.6% vs. 3.5%; p = 0.33), and prolonged postoperative pain (1.8% vs. 4.1%; p = 0.12). No differences were observed between traumatic and nontraumatic tears.

Conclusion

RCR within three months of imaging is associated with statistically lower one-year pain scores but no clinically meaningful differences in outcomes or complications.

## Introduction

Chronic shoulder pain affects around 8% of all American adults, ranking second only to chronic knee pain among musculoskeletal diseases [1]. Rotator cuff tears account for about 40% of shoulder dysfunction and 30%-40% of cases of shoulder pain in all patients [2]. They are often preceded by rotator cuff tendonitis and most commonly involve the supraspinatus tendon [3]. The decision to pursue conservative versus operative treatment is multifactorial and depends on the etiology, duration, and tear size [4,5]. Surgical repair is considered for patients who have an acute traumatic or chronic full-thickness tear. Rotator cuff tears that result in significantly reduced arm function, show insufficient improvement after 3-6 months of conservative treatment, or possess characteristics that make them high risk of progression may also warrant surgical intervention. Aside from the known benefits of early intervention for acute, traumatic tears, the effect that timing of diagnostic imaging has on surgical outcomes remains under investigation. While some studies suggest early repair (4-6 months from diagnostic imaging) of acute traumatic rotator cuff tears yields better clinical outcomes including lower re-rupture rates, faster recovery, successful return to sports, and reduced need for advanced interventions like grafting, others report no significant impact on outcomes, such as pain, reoperation rates, or range of motion, even with delays in surgery up to a year for both traumatic and chronic tears [6-17]. Given these mixed findings, the primary aim of this retrospective study was to evaluate whether RCR performed within three months of diagnostic imaging was associated with differences in postoperative pain, range of motion, and patient-reported outcomes compared with repair performed after three months. A three-month threshold was selected based on prior studies evaluating early versus delayed rotator cuff repair (RCR) using a similar interval and to provide a clinically interpretable definition of surgical delay [14]. Secondary objectives were to compare complication rates between timing cohorts and determine whether these associations differed between traumatic and degenerative tears. We hypothesized that earlier repair would be associated with improved postoperative outcomes.

## Materials and methods

Methods

A retrospective review of patients in a single health system who underwent RCR with 15 fellowship-trained surgeons at a large metropolitan hospital system from 2014 to 2022 was conducted. The IRB approved this study (13963). Patients were stratified based on the timing of surgery relative to the timing of diagnostic magnetic resonance imaging (MRI) or ultrasonography (US). Patient cohorts included those 18 years and older who had an imaging date <3 months or ≥3 months from their surgical date. The timing of the surgical treatment was determined by the operating surgeon, without a specific protocol in place. Patients who had previous rotator cuff surgery, concomitant shoulder surgery including shoulder arthroplasty, and those who had insufficient follow-up were excluded from the study. Patient demographics, visual analog scale (VAS) pain scores, active range of motion in forward flexion, abduction, and external rotation were measured preoperatively and postoperatively at intervals of one week, six weeks, six months, one year, and two years. All patients with available data points were averaged at each of the respective time intervals. Patient-reported outcomes of function and pain were collected using the Patient-Reported Outcomes Measurement Information Survey (PROMIS) system and were collected at the same time intervals using PROMIS upper extremity (UE) function and pain interference (PI) [17,18]. Complication rates, including adhesive capsulitis, re-tears, and unresolved chronic pain, were collected. A subanalysis was performed between traumatic and nontraumatic rotator cuff tears. A traumatic rotator cuff tear was characterized as a tear on MRI or US in patients who experienced a discrete injury to the shoulder that triggered symptoms without prior complaints. Degenerative tears were characterized as chronic tears that were either symptomatic or asymptomatic and present for more than six weeks according to the patient’s reported history. These cohorts were then further stratified into subgroups by timing from imaging to surgery to perform statistical analysis.

Statistical methods

Results were analyzed using Chi-square and unpaired t-test correlations with significance determined at p < 0.05. Statistics were calculated using IBM SPSS Statistics for Windows, Version 29 (Released 2022; IBM Corp., Armonk, New York, United States). A priori sample-size calculation was not used to determine enrollment, as sample sizes were substantially smaller for several outcomes, particularly PROMIS measures.

## Results

A total of 487 patients were included in the study, with 342 patients in the <3 months between MRI or US and surgery cohort and 145 patients in the >3-month cohort. There was no significant difference in age, race, body mass index, area deprivation index, or laterality of tears. There was a significant difference in sex, with the <3-month cohort having 187 males (55%) and 155 females (45%), while the >3-month cohort had 54 males (37%) and 91 females (63%) (p = 0.04). Male patients were significantly more likely to wait fewer than three months for surgery, while female patients were significantly more likely to wait at least 3 months for surgery (p = <0.01) (Table 1).

**Table 1:** Patient demographics n, sample size; SD, standard deviation; χ2 = Chi-square; * indicates p-value of <0.05

Demographic	<3 months mean ± SD (n = 342)	>3 months mean ± SD (n = 145)	p-value	t-value (t), Chi-square (χ2)
Age (years), mean ± SD	56.87 ± 9.27	57.41 ± 9.15	0.55	t = 0.59
Body mass index (kg/m2), mean ± SD	30.7 ± 6.1	31.36 ± 7.45	0.31	t = 1.02
Sex, n (%)			<0.01*	χ2 = 12.39
Male	187 (54.7%)	54 (37.2%)		
Female	155 (45.3%)	91 (62.8%)		
Race, n (%)			0.41	χ2 = 4.00
White	229 (67.0%)	86 (59.3%)		
Black	95 (27.8%)	53 (36.6%)		
Hispanic	7 (2.0%)	3 (2.1%)		
Asian	3 (0.9%)	1 (0.7%)		
Other	8 (2.3%)	2 (1.4%)		
Area Deprivation Index, mean ± SD	64.49 ± 25.4	68.16 ± 25.2	0.14	t = 1.46
Laterality, n (%)			0.54	χ2 = 0.37
Left	119 (34.8%)	46 (31.7%)		
Right	223 (65.2%)	98 (67.6%)		

There was a significant difference in preoperative forward-flexion active range of motion between patients who had MRI or US <3 months before surgery (126.34 ° ± 42.41°) and patients who had MRI or US >3 months before surgery (134.85° ± 33.82°) (p = 0.039). There was also a significant difference in PROMIS UE between the <3-month group (33 ± 6.52) and >3-month group (26.2 ± 4.02) (p = 0.047) (Table 2).

**Table 2:** Postoperative clinical and patient-reported measures ABD, abduction; AROM, active range of motion; ER, external rotation; FF, forward flexion; PROMIS PI, Patient-Reported Outcomes Measurement Information System Pain Interference; PROMIS UE, Patient-Reported Outcomes Measurement Information System Upper Extremity; n, sample size; SD, standard deviation; VAS, visual analog scale; * indicates p-value of <0.05

Follow-up period		<3 months mean ± SD	>3 months mean ± SD	p-value	t-value
1-week follow-up	VAS	4.73 ± 2.53 (n = 214)	4.96 ± 2.63 (n = 95)	0.45	0.73
	PROMIS UE	26.29 ± 6.21 (n = 7)	19.67 ± 4.62 (n = 3)	0.14	1.64
	PROMIS PI	66.33 ± 3.16 (n = 9)	62.67 ± 5.51 (n = 3)	0.17	1.46
6-week follow-up	VAS	4.01 ± 2.61 (n = 215)	4.66 ± 2.65 (n = 94)	0.044*	2.00
	FF AROM	99.87 ± 41.38 (n = 104)	102.19 ± 43.01 (n = 32)	0.78	0.27
	ABD AROM	82.44 ± 34.5 (n = 81)	87.10 ± 42.98 (n = 20)	0.61	0.51
	ER AROM	34.87 ± 19.87 (n = 110)	47.52 ± 14.38 (n = 33)	0.001*	3.40
	PROMIS UE	30 ± 5.04 (n = 15)	25.6 ± 65.50 (n = 14)	0.80	0.26
	PROMIS PI	62.80 ± 5.72 (n = 15)	61.25 ± 4.86 (n = 8)	0.52	0.65
3-month follow-up	VAS	3.355 ± 2.66 (n = 221)	3.72 ± 2.63 (n = 91)	0.27	1.09
	FF AROM	125.85 ± 81.86 (n = 279)	121.58 ± 32.89 (n = 116)	0.59	0.54
	ABD AROM	94.40 ± 33.40 (n = 159)	97.40 ± 34.40 (n = 55)	0.57	0.57
	ER AROM	41.70 ± 18.82 (n = 255)	39.52 ± 17.96 (n = 104)	0.31	1.00
	PROMIS UE	35.57 ± 6.57 (n = 14)	29.00 ± 7.72 (n = 6)	0.067	1.95
	PROMIS PI	57.95 ± 5.58 (n = 19)	58.30 ± 6.00 (n = 10)	0.88	0.16
6-month follow-up	VAS	2.91 ± 2.67 (n = 216)	3.05 ± 2.53 (n = 88)	0.68	0.42
	FF AROM	142.07 ± 29.70 (n = 268)	145.22 ± 25.98 (n = 120)	0.32	1.00
	ABD AROM	107.36 ± 35.78 (n = 152)	112.66 ± 38.17 (n = 67)	0.32	0.99
	ER AROM	47.16 ± 17.3 (n = 247)	49.3 ± 18.46 (n = 114)	0.29	1.07
	PROMIS UE	41.2 ± 7.08 (n = 15)	30.86 ± 8.97 (n = 7)	0.01*	2.94
	PROMIS PI	54.83 ± 7.69 (n = 18)	57.27 ± 7.76 (n = 11)	0.42	0.83
1-year follow-up	VAS	2.66 ± 2.66 (n = 242)	3.31 ± 3.31 (n = 103)	0.046*	1.93
	FF AROM	149.32 ± 28.77 (n = 308)	151.53 ± 23.13 (n = 134)	0.44	0.79
	ABD AROM	114.97 ± 38.03 (n = 183)	118.01 ± 38.47 (n = 73)	0.57	0.58
	ER AROM	50.87 ± 19.42 (n = 277)	52.88 ± 20.39 (n = 120)	0.35	0.93
	PROMIS UE	37.94 ± 9.6 (n = 16)	34.88 ± 7.99 (n = 8)	0.45	0.78
	PROMIS PI	56.50 ± 8.33 (n = 20)	56.18 ± 7.08 (n = 11)	0.92	0.11
2-year follow-up	VAS	5.19 ± 2.69 (n = 27)	5.45 ± 1.51 (n = 11)	0.75	0.30
	FF AROM	137.65 ± 36.33 (n = 34)	144.17 ± 24.39 (n = 18)	0.50	0.68
	ABD AROM	113.18 ± 38.10 (n = 22)	110.00 ± 28.28 (n = 2)	0.91	0.11
	ER AROM	46.43 ± 18.6 (n = 28)	46.43 ± 16.81 (n = 14)	1.00	0.00

There was a significant decrease in pain scores at six-week follow-up in patients who had MRI or US within three months of surgery (4.01 ± 2.61) versus patients who waited longer than three months to have surgery following imaging (4.66 ± 2.65) (p = 0.04). There was also a significant increase in PROMIS UE function scores in patients who waited at least three months between MRI/US and surgery at six-month follow-up (41.2 ± 7.08) compared to patients who waited <3 months (30.86 ± 8.97) (p = 0.01). However, there was limited patient data for this metric, which may impact the significance within the overall cohort. Finally, there was a significant decrease in VAS pain scores in patients who had MRI/US within three months of their surgery at one-year follow-up (2.66 ± 2.66) compared to the >3-month cohort (3.31 ± 3.31) (p = 0.046). All other data points had no significant difference between the cohorts (Table 3).

**Table 3:** Preoperative clinical and patient-reported measures ABD, abduction; AROM, active range of motion; ER, external rotation; FF, forward flexion; PROMIS PI, Patient-Reported Outcomes Measurement Information System Pain Interference; PROMIS UE, Patient-Reported Outcomes Measurement Information System Upper Extremity; n, sample size; SD, standard deviation; VAS, visual analog scale.* indicates p-value of <0.05

Preoperative period	Outcome measure	<3 months mean ± SD	>3 months mean ± SD	p-value	t-value
	VAS	6.36 ± 2.3 (n = 241)	6.48 ± 2.31 (n = 102)	0.67	0.44
	Forward flexion AROM	126.34 ± 42.41 (n = 316)	134.85 ± 33.82 (n = 135)	0.039*	2.07
	Abduction AROM	99.34 ± 38.01 (n = 182)	103.66 ± 33.48 (n = 67)	0.41	0.82
	External rotation AROM	49.86 ± 20.38 (n = 295)	50.3 ± 18.7 (n = 117)	0.84	0.20
	PROMIS UE	33 ± 6.52 (n = 13)	26.2 ± 4.02 (n = 5)	0.047*	2.16
	PROMIS PI	62.82 ± 5.5 (n = 17)	63.17 ± 3.97 (n = 6)	0.89	0.14

Subanalysis comparison of traumatic and nontraumatic tear etiology demonstrates there was no significant difference in postoperative outcomes between patients who had traumatic etiologies for their rotator cuff tears versus those who had a chronic etiology at one-year follow-up when comparing timing from MRI/US to RCR surgery (Table 4).

**Table 4:** Complication rates following rotator cuff repair n, sample size

Complications		>3 months (n = 145)	Chi-square	p-value
N (%)	59 (17.26%)	29 (20.0%)	0.52	0.47
Re-tears				
N (%)	32 (9.36%)	17 (11.72%)	0.63	0.43
Adhesive capsulitis				
N (%)	19 (5.59%)	5 (3.45%)	0.97	0.33
Biceps tear				
N (%)	6 (1.75%)	6 (4.14%)	2.41	0.22

When considering the rate of complications following RCR in both cohorts, 17% of patients in the <3-month to RCR cohort reported complications compared to 20% in the ≥3-month cohort, but there was no significant difference in overall rate (p = 0.43). A subanalysis of these complications between the early and delayed groups was performed, looking at the rate of rotator cuff re-tears (9.36% vs. 11.72%; p = 0.43), adhesive capsulitis (5.56% vs. 3.45%; p = 0.33), and biceps rupture (1.75% vs. 4.41%; p = 0.22), but found no significant difference between cohorts (Table 5).

**Table 5:** Subanalysis between traumatic and chronic rotator cuff tears at one-year follow-up ABD, abduction; AROM, active range of motion; ER, external rotation; FF, forward flexion; PROMIS PI, Patient-Reported Outcomes Measurement Information System Pain Interference; PROMIS UE, Patient-Reported Outcomes Measurement Information System Upper Extremity; n, sample size; SD, standard deviation; VAS, visual analog scale

	<3 months mean ± SD	>3 months mean ± SD	p-value	t-value
Traumatic tear (n = 297)				
VAS	2.67 ± 2.62 (n = 150)	3.32 ± 3.30 (n = 57)	0.14	1.48
FF AROM	148.94 ± 30.22 (n = 186)	151.58 ± 23.15 (n = 76)	0.49	0.68
ABD AROM	113.67 ± 39.91 (n = 113)	116.31 ± 38.43 (n = 42)	0.71	0.37
ER AROM	51.27 ± 20.57 (n = 166)	53.66 ± 21.52 (n = 67)	0.43	0.79
PROMIS UE	38.77 ± 10.03 (n = 13)	38.00 ± 8.89 (n = 3)	0.9	0.12
PROMIS PI	56.23 ± 9.37 (n = 13)	55.00 ± 10.12 (n = 5)	0.81	0.24
Degenerative tear (n = 190)				
VAS	2.68 ± 2.79 (n = 87)	3.22 ± 2.66 (n = 41)	0.30	1.04
FF AROM	150.83 ± 26.26 (n = 115)	151.47 ± 24.03 (n = 51)	0.88	0.15
ABD AROM	117.12 ± 35.43 (n = 66)	122.41 ± 39.52 (n = 29)	0.52	0.65
ER AROM	50.19 ± 18.1 (n = 104)	51.38 ± 19.27 (n = 47)	0.71	0.37
PROMIS UE	34.33 ± 8.02 (n = 3)	33.00 ± 7.78 (n = 5)	0.82	0.23
PROMIS PI	56.17 ± 6.79 (n = 6)	57.17 ± 3.97 (n = 6)	0.76	0.29

## Discussion

Our study evaluated the timing of arthroscopic RCR and its impact on functional and patient-reported outcomes. We hypothesized that early intervention (within three months of diagnostic imaging) would result in improved outcomes compared to delayed surgery. However, our findings showed that patients who underwent RCR within three months had similar outcomes to those who received surgery after three months, with no significant differences in functional scores or patient-reported measures.

With respect to pain, as measured by the VAS, the early intervention group demonstrated statistically significant reductions at both six weeks (4.01 ± 2.61 vs. 4.66 ± 2.65, p = 0.044) and one-year follow-up (2.66 ± 2.66 vs. 3.31 ± 3.31, p = 0.0463) compared to the delayed group. However, these differences did not meet the established minimal clinically important difference of 1.4 [19]. These results are consistent with those of a recent prospective study conducted during the COVID-19 pandemic by Clinker et al., which observed delayed treatment of degenerative rotator cuff tears by three months. Despite early improvements in American Shoulder and Elbow Surgeons, Simple Shoulder Test, and VAS scores, this study reported no long-term difference between early (<3 months) and delayed repair groups at two-year follow-up [20]. Notably, the study population was relatively small (n = 30), and all surgeries were performed by two orthopedic surgeons from the same institution using the same repair technique. In contrast, our study analyzed outcomes from a larger cohort (n = 487), with procedures performed by more than 10 orthopedic surgeons utilizing different techniques from the same institution, thereby strengthening the generalizability of these findings.

When evaluating functional outcomes, PROMIS scores, and complication rates, our results align with previous studies suggesting that surgical delay for both traumatic and degenerative tears of up to one year does not significantly impact outcomes such as VAS, range of motion, subjective shoulder value, reoperation rates, or patient-reported outcome measures [11,13-16]. Notably, we observed that delaying RCR beyond three months was not associated with higher rates of complications such as adhesive capsulitis, rotator cuff re-tears, or biceps rupture. These findings are consistent with a meta-analysis by van der List et al. that included 8,118 patients across 33 studies and found no increase in re-tear rates with surgical delays under one year. However, the same study demonstrated that delays of one year or more were associated with significantly higher re-tear rates [20,21]. This is supported by additional data from Clinker et al. showing a 27% increase in failure at two-year follow-up among patients who underwent delayed RCR, although this difference was only numerically significant and not statistically significant (p = 0.20) [20]. Patel et al. also found that the early repair group experienced a significantly shorter time to full recovery (14 vs. 33.8 months; p = 0.05) [11]. Other studies have reported better functional outcomes and lower re-tear rates (9.9%) with early repair, while repairs delayed beyond 12 months were associated with increased revision risk (15.2%) at five-year follow-up [12]. These discrepancies highlight the need for further long-term, prospective studies to define optimal surgical timing more precisely.

Our findings remained consistent when stratifying patients by mechanism of injury. Approximately 60.8% of injuries in our cohort were traumatic, while 39.1% were degenerative. The lower proportion of degenerative injuries may be due to the insidious onset of symptoms and variability in pain perception, often leading patients to delay care until daily functioning is significantly impaired. Importantly, even in patients who waited over six months for RCR, regardless of traumatic or chronic etiology, there were no significant differences in postoperative outcomes. These results challenge the conventional approach favoring early surgery in traumatic tears and support the consideration of conservative management strategies, particularly in patients with higher surgical risk or lower functional demands [22]. In further support of conservative treatment, recent 10-year data from the MOON Shoulder group demonstrated that most patients with symptomatic, atraumatic full-thickness rotator cuff tears treated nonoperatively with physical therapy experienced lasting improvement and did not transition to surgical intervention [23]. These findings further strengthen the argument for a more individualized approach to surgical timing.

This study has several limitations. First, its retrospective design introduces the potential for selection bias, missing data, and residual confounding and prevents causal inference regarding surgical timing. Surgical timing was determined through routine clinical decision-making rather than a standardized protocol, and unmeasured patient-, surgeon-, or system-level factors may therefore have influenced assignment to the timing cohorts. Significant baseline differences were present between groups, including sex distribution and preoperative forward-flexion range of motion, which may confound unadjusted postoperative comparisons. Tear morphology, including tear size, tendon retraction, and degree of fatty infiltration, was not consistently available in a standardized format and could not be controlled for. Surgical techniques and postoperative rehabilitation protocols were also not standardized across surgeons. Additionally, the etiology of rotator cuff tears can be difficult to classify reliably because traumatic tears may occur in previously degenerative tendons and degenerative tears may become symptomatic after an unrecognized injury. Finally, PROMIS outcomes were available for relatively small subsets of patients, particularly at later follow-up intervals, and loss to follow-up and inconsistent completion of patient-reported outcome surveys may introduce attrition bias. These limitations should be considered when interpreting the observed associations.

## Conclusions

In this retrospective cohort of patients undergoing RCR, repair within three months of diagnostic imaging was associated with statistically lower VAS pain scores at selected postoperative intervals; however, these differences did not meet the established minimal clinically important difference (MCID). Most measured functional outcomes and complication rates did not significantly differ between timing cohorts. Within the limitations of this observational study, including potential selection bias, baseline differences, residual confounding, and incomplete patient-reported outcome data, a delay beyond three months from diagnostic imaging to RCR was not associated with clinically meaningful differences in the measured postoperative outcomes. Prospective studies controlling for tear morphology, patient characteristics, surgical technique, and rehabilitation are needed to further define the relationship between surgical timing and outcomes.
